# Glucose and Lactate Transport in Pancreatic Cancer: Glycolytic Metabolism Revisited

**DOI:** 10.1155/2018/6214838

**Published:** 2018-12-04

**Authors:** Miles E. Cameron, Anastasiya Yakovenko, Jose G. Trevino

**Affiliations:** Department of Surgery, University of Florida Health Sciences Center, Gainesville, FL 32610, USA

## Abstract

Membrane transporters fulfill essential roles in maintaining normal cellular function in health. In cancer, transporters likewise facilitate the aberrant characteristics typical of proliferating tumor cells. Pancreatic ductal adenocarcinoma is remarkable in its aggressiveness, and its metabolism is supported by a variety of membrane transporters. Glucose transporter 1 is upregulated in pancreatic cancer, enables rapid cellular uptake of glucose, and contributes to the invasiveness and metastatic ability of the disease. Likewise, the machinery of glycolysis, enzymes such as pyruvate kinase type M2 and hexokinase 2, is particularly active and ultimately leads to both lactate and tumor formation. Lactic acid channels and transporters include monocarboxylate transporters 1 and 4, connexin43, and CD147. In conjunction with glucose transporters and glycolytic metabolism, lactic acid transport helps perpetuate tumor cell metabolism and contributes to the formation of the unique tumor microenvironment in pancreatic cancer. These transporters may serve as potential therapeutic targets.

## 1. Introduction

Pancreatic ductal adenocarcinoma (PDAC) is a horrible disease with a five-year survival of 8% [1]. In spite of ongoing research, there have been few clinical improvements over the past 50 years. One familiar observation in many cancers, including PDAC, is a profound reprogramming of cellular metabolism. When this reprogramming involves a transition to aerobic glycolysis, it is commonly referred to as the Warburg effect. As with all cells, energy in the form of ATP is derived from glucose. Tumors exhibiting the Warburg effect however do not fully oxidize glucose to its potential. This feature has recently received renewed attention in the literature. Accordingly, this review focuses specifically on the metabolite transporters that are active in PDAC, namely, the conduits of glucose and lactic acid. Their roles in facilitating abnormal metabolism, formation of the tumor microenvironment, and possible therapeutic implications are discussed.

## 2. Glycolysis and Glucose Transporters

It has been well known for decades that malignant cells require high levels of glucose compared to normal cells. In a phenomenon first described by Otto Warburg in the 1920s, glycolysis is often the dominant source of ATP, even in oxygen-rich conditions [2]. This is now known as the Warburg effect, and it characterizes the use of inefficient metabolism by cancer cells. To further emphasize the issue, metabolic reprogramming was recognized as an emerging “hallmark of cancer” by Hanahan and Weinberg in 2011 [3]. The possible advantages of this reprogramming have been extensively discussed, but the Warburg effect and other characteristic phenotypes of cancer metabolism are not completely understood. There must be benefits in addition to ATP production that cancer cells receive from what are otherwise inefficient means. Glycolytic intermediates serve as precursors of nucleotides, phospholipids, and other biomolecules via the pentose phosphate pathway [4]. Thus, the accumulation of biomass and formation of solid tumors is central to the role of cancer cells. Indeed, it appears that abnormal metabolism may in itself drive cancer progression through a preference for enzymatic isoforms that favor anabolism [5]. For instance, pyruvate kinase type M2 and hexokinase 2, key enzymes in the glycolytic pathway of PDAC cells, contribute to the invasive potential and metastatic ability of the tumor [6, 7]. Dysregulated metabolism further manifests itself in several ways that contribute to a “cancer syndrome” in which systemic metabolism is deleteriously affected leading to specific cancer-induced muscle and adipose wasting, or cachexia [8]. The mechanisms leading to cachexia in PDAC are complex and varied, but dysregulated metabolism plays a role. Cachexia is a particularly burdensome facet of the disease and contributes greatly to mortality [9]. To similar deleterious effect, the use of glycolysis eliminates radical oxygen species that are generated during oxidative phosphorylation [10, 11]. This serves to preserve the genotype of viable tumor cells.

Glucose transporters (GLUTs), which belong to the solute carrier (SLC2A) family of transport proteins, are known to mediate glucose uptake by cells [12]. GLUTs are present in many tissues across the body and facilitate a number of roles. GLUT-1 has been most extensively studied in PDAC; it is thus the principal focus of this review. GLUT-1 is expressed in tissues with high glucose demand that principally undergo glycolysis. This includes normal cells that display the Warburg effect, such as hematopoietic stem cells [13, 14]. GLUT-1 thereby mitigates the first rate-limiting step in metabolism which is the transmembrane transport of glucose [15]. While GLUT-1 is responsible for basal glucose uptake in high-requirement cells, it plays additional roles central to both tumorigenesis and hypoxia [16]. GLUT-1 provides much needed substrate to tumor cells that express the Warburg phenotype in the presence of oxygen. GLUT-1 likewise provides glucose for cells to perform anaerobic glycolysis in hypoxic environments. Such is the case in PDAC: tumors are characterized by both acidity and hypoxia and, in distinct portions, hyperperfusion driven by neoangiogenesis [17].

## 3. Gene Expression in the Hypoxic Tumor Microenvironment

Solid tumors have the potential to thrive in otherwise hypoxic microenvironments by means of metabolism not seen in normal physiology. There are several factors that beneficially follow a localized hypoxia that protect the cell from stress and promote tumor growth, including the hypoxia-inducible factors (HIFs). In PDAC, HIF-1alpha functions to upregulate proteins that are essential to survival in oxygen-deprived states [18]. HIF-1alpha has been extensively studied for many years and directly activates the transcription of GLUTs, enzymes essential to tumor cell glycolysis, vascular endothelial growth factor (VEGF) and other proteins essential to cellular proliferation [19]. Notably, an increased level of HIF-1alpha is specifically associated with increased expression of GLUT-1 that allows the aforementioned flux of glucose down its concentration gradient [20]. In addition to HIF-1alpha, increased expression of sirtuin 1, a lysine deacetylase with several enzymatic targets, has been implicated both in increased GLUT-1 expression and the glycolytic reprogramming of pancreatic tumor cells [21]. In a similar manner, GLUT-3 is upregulated by tumor cells under hypoxic stress [22]. The expression of GLUTs is also induced by the Ras and Src signaling pathways, both of which are canonical mechanisms of pancreatic oncogenesis [23]. There are two opposing explanations accounting for the high expression of GLUT-1 that illustrate its role in hypoxic and oncogenic pathways (or both combined due to secondary changes to the microenvironment): (1) glucose is used in excess via glycolytic pathways leading to intracellular depletion of glucose and the subsequent recruitment of facilitative transport enables a sustained high metabolic rate, and (2) the increased expression of GLUT-1 allows accelerated glycolysis by increasing intracellular glucose concentrations beyond normal physiological limits. Both mechanisms explain the high level of GLUT-1 found in response to upregulation and the Warburg effect in simplistic terms. In clear cell variants of PDAC, GLUT-1 is associated with cytoplasmic glycogen accumulation supporting the later hypothesis [24]. Clear cell features are however only observed in 24% of studied PDAC tumors [25]. GLUT-1 expression is also dependent on insulin levels, and the incidental insulin resistance frequently observed in PDAC may contribute to and propagate a glycolytic phenotype [26].

**Figure 1:**
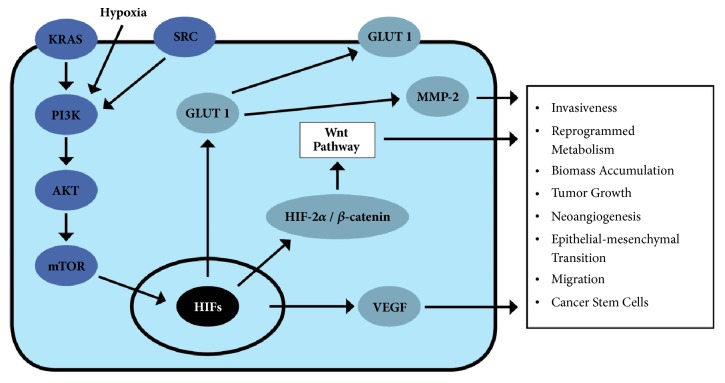
**Several signaling pathways lead to the expression of GLUT-1 in PDAC cells**. These include canonical Ras and Src pathways in addition to the adaptive response to a hypoxic microenvironment. Other factors associated with GLUT-1 expression are represented that characterize the aberrant physiology of tumor cells.

In either case, the ability of pancreatic tumors to overexpress glucose transporters corresponds to the other actions of transcription regulators in the hypoxic microenvironment that is the upregulation of glycolysis enzymes. Key checkpoint enzymes such as pyruvate dehydrogenase kinase 1 and lactate dehydrogenase A are controlled when HIF-1alpha is silenced, confirming that HIF-1alpha mediates the transcription of numerous proteins in addition to GLUT-1 [27]. Additionally, when HIF-1alpha and HIF-2alpha are suppressed, there is a decrease in glucose utilization and lower lactic acid production [28]. This corresponds to an attenuation of glycolysis. Furthermore, it has been noted that suppression of HIF-1alpha and HIF-2alpha directly inhibits tumor growth, invasion ability, and migration of pancreatic cancer cells [27, 28]. HIF-2alpha, which has not been as thoroughly investigated as HIF-1alpha, has been found to interact with beta-catenin and form a particularly stable conformation that specifically promotes the epithelial-to-mesenchymal transition and stem cell qualities of certain tumor cells via the Wnt signaling pathway [29]. As these factors induced by hypoxia contribute profoundly to the invasiveness of cancer, their role as prognostic markers has likewise been considered (see Figure 1).

## 4. Histological Markers of Hypoxia

Increased expression of HIF-1alpha and sirtuin 1 is associated with poorer overall survival in PDAC [20, 24]. Both result in increased GLUT-1. Mechanistically, forced overexpression of GLUT-1 as a result of hypoxia-mediated transcription increases the activity of matrix metalloproteinase 2 (MMP-2) which hastens the development of invasive PDAC [30]. HIFs also lead to downregulation of tryptophanyl-tRNA synthetase (TrpRS) which has been studied in both colorectal and pancreatic cancers for its significance in prognosis [31]. TrpRS is downregulated in hypoxia and may function in the development of tumors with high metastatic ability. In addition to increasing GLUT-1 levels, sirtuin 1 is associated with the loss of* cell cycle and apoptosis regulator 2* (*CCAR2*) which accordingly stimulates unchecked growth and proliferation [21].

In spite of these findings, there is significant controversy in recent literature surrounding the prognostic relationship between GLUT-1 and PDAC. Most sources suggest that high GLUT-1 expression on immunohistochemical assays of resected tumors corresponds to a tumor size greater than 2cm (T2 and greater), nodal involvement (N1), and shorter overall patient survival [32]. Profound GLUT-1 increases have also been correlated positively to histological grade in many cancers of the biliary system, including cancers of the gallbladder, bile duct, and ampulla of Vater [24, 33]. Cellular proliferation, as measured by Ki-67, is also related positively to GLUT-1 expression [34]. However, others find correlations only between GLUT-1 and poor overall survival [35]. Nevertheless, there is concord that both GLUT-1 and GLUT-3 may serve as a prognostic marker [36]. The literature is similarly divided with regard to 18F-fluoro-2-deoxyglucose (FDG) uptake in positron emission tomography/computed tomography (PET/CT); some findings correlate increased maximal standardized uptake value to GLUT-1 expression [34, 35, 37, 38]. The lack of validated proportionality is surprising given the nature of FDG uptake in PET/CT evaluation [39]. Other families of glucose transporters, such as the sodium-dependent glucose transporters (SGLTs) have also been studied in tumors using immunohistochemical mapping [40]. Notably, SGLT-2 is expressed in PDAC cells; this has immediate implication due to the possible use of SGLT inhibitors in preventing further cellular glucose uptake.

## 5. Lactate Transport and Tumor Stroma

In the absence of oxidative phosphorylation, it follows that glycolytic cancer cells with metabolism driven by the facilitated transport of glucose would necessitate a corresponding facilitated release of glycolysis' end product, lactic acid. One mechanism couples proton and lactate efflux via monocarboxylate transporters (MCTs) [41]. The MCT family plays a significant role in many body tissues, including red blood cells, T-lymphocytes, white muscle, and tumor cells; all tissues that typically undergo glycolysis in aerobic conditions [42]. In particular, MCT-1, the most ubiquitous of the MCT isoforms across all tissue types, assists in restoring the pH of cells undergoing glycolysis through proton efflux [43]. The expression of MCT-1 parallels that of GLUT-1 with regard to tissue localization. The coordination of MCTs is furthermore useful in tumorigenesis, as the more glycolysis-driven centers of solid tumors can with transport rapidly provide the periphery—which has been superiorly perfused via neoangiogenesis secondary to VEGF expression in hypoxia—with substrate (lactate) for complete oxidation via electron transport and oxidative phosphorylation [44]. Studies have specifically associated MCT-4 to pancreatic cancer cell migration, and MCT-1 and MCT-4 have been linked to invasion ability [45]. Recently CD147—also known as basigin—has been identified as a regulator of several transporter proteins, including the MCT-1 and MCT-4, and that CD147 expression is mediated by the synthesis of matrix metalloproteins in tumor-associated fibroblasts [46, 47]. Indeed, tumorigenicity is inhibited in vivo when CD147 expression is silenced through RNA interference by increasing the intracellular concentration of lactate through inhibition of MCT-1 and MCT-4 expression in cells bearing a Warburg phenotype [48]. Of further note, CD147 has also been associated with glutamine transport and calcium signaling, and thus may serve as an ideal therapeutic target due to its role in multiple metabolic pathways [46, 47].

When studied via gap junctions, lactate is more rapidly discharged from cancer cells than protons, which are highly-buffered by a variety of intracellular bases [41]. Extracellular lactate thus was found to produce an alkalinized rim directly surrounding the pericellular space. This microenvironment at this rim of tumor cells further favors tumor growth, particularly when the intermediates of aerobic glycolysis provide the building blocks of tumor biomass [10, 41]. Connexin43 (Cx43), a component of competent gap junctions in the pancreas, has been identified as an important conduit for lactate, stabilizing and functioning as a component of intercellular gap junctions [41]. By serving as a channel, Cx43 facilitates the alkalization of proliferating tumor cells in an otherwise acidic environment. Lactate is sequestered away from hypoxic microscopic foci in the developing tumor to better perfused recipient cells where it may serve as a substrate for oxidative phosphorylation; this schema of metabolite sequestration parallels the actions of MCT-1 and MCT-4 described previously. The utilization of lactate as substrate for further oxidation is a third benefit of the Warburg effect [10]. These actions of Cx43 allow for the dynamic interplay between glycolysis and oxidative phosphorylation that enables pancreatic cancer cells to thrive [49].

Besides serving as a channel for lactate dissipation, Cx43 mediates cellular communication between cancer and tumor-associated stromal cells. For example, pancreatic stellate cells express Cx43 and type 1 collagen in proliferation [50]. The dysregulation of Cx43 is also well known to parallel the progression of pancreatic cancer. In pancreatic intraepithelial neoplasia (PanIN), Cx43 is localized to the basolateral membrane of pancreatic duct cells [51]. As tumorigenesis progresses, Cx43 becomes more associated with the surrounding stroma. Cx43 normally exists in different states of phosphorylation [52]. These phosphoisoforms are believed to reflect the localization of Cx43, patency of the gap junction, and ability to participate in epithelial-stromal communication [51]. Extracellular signal-related kinase (ERK) modulates Cx43 localization and contributes to malignancy in response to growth factor stimulation [53]. To further characterize the relationship, ERK and various epidermal growth factors are expressed at increased levels in PanINs [54]. In contrast to these findings, advanced pancreatic tumors and cancer stem cells typically do not express Cx43, and gap junction function is interrupted [55]. These changes in the competency of gap junctions has been implicated in therapy, both in regard to novel approaches and in consideration of mechanisms underlying the resistance to conventional chemotherapy.

## 6. Conclusion

Transporters play a facilitative role in the metabolism that characterizes PDAC tumor cells. There is much evidence that the overexpression of glucose transporters and glycolytic enzymes positively influence tumor growth and increase mortality. The alterations in glucose handling by tumor cells may represent a systemic cancer syndrome that contributes to the profound symptoms of aggressive tumors such as found in PDAC. Of note, the discharge and localization of lactic acid are necessary for tumorigenesis by creating an ideal tumor microenvironment. There are multiple transporters involved in the reprogrammed metabolism PDAC, including GLUT-1, MCT-1 and -4, CD147, and Cx43; all may serve as potential therapeutic targets, particularly when combined with standard therapies.
